# An Alternative Wearable Tracking System Based on a Low-Power Wide-Area Network

**DOI:** 10.3390/s17030592

**Published:** 2017-03-14

**Authors:** Raul Fernández-Garcia, Ignacio Gil

**Affiliations:** Department of Electronic Engineering, Universitat Politècnica de Catalunya, Colom 1, 08222 Terrassa, Spain; ignasi.gil@upc.edu

**Keywords:** wearable, wireless sensor node, textile antenna, GPS, SIGFOX, SENTILO

## Abstract

This work presents an alternative wearable tracking system based on a low-power wide area network. A complete GPS receiver was integrated with a textile substrate, and the latitude and longitude coordinates were sent to the cloud by means of the SIM-less SIGFOX network. To send the coordinates over SIGFOX protocol, a specific codification algorithm was used and a customized UHF antenna on jeans fabric was designed, simulated and tested. Moreover, to guarantee the compliance to international regulations for human body exposure to electromagnetic radiation, the electromagnetic specific absorption rate of this antenna was analyzed. A specific remote server was developed to decode the latitude and longitude coordinates. Once the coordinates have been decoded, the remote server sends this information to the open source data viewer SENTILO to show the location of the sensor node in a map. The functionality of this system has been demonstrated experimentally. The results guarantee the utility and wearability of the proposed tracking system for the development of sensor nodes and point out that it can be a low cost alternative to other commercial products based on GSM networks.

## 1. Introduction

In recent years, the electronic semiconductors industry growth has allowed a reduction in the cost of electronic products and an increase in wireless communication applications. Due to this fact, nowadays, several examples of low cost electronic products with wireless interfaces have been introduced to the market. Among them, a massive increase of wireless sensor networks (WSN) has been produced in industrial and domestic applications, helping to develop the internet of things revolution. The WSN consist of small communication nodes. These nodes contain a sensing part, a microcontroller, communication components and power supply (typically a battery). These nodes should fulfil restrictions of low-cost and low-power consumption to guarantee their usefulness. In this sense, some references can be found in the literature focused on increasing battery lifetimes by means of codified algorithms [1], reducing transmission requirements, or proposing additional energy sources, such as radiofrequency (RF) energy harvesting [2].

**Figure 1:**
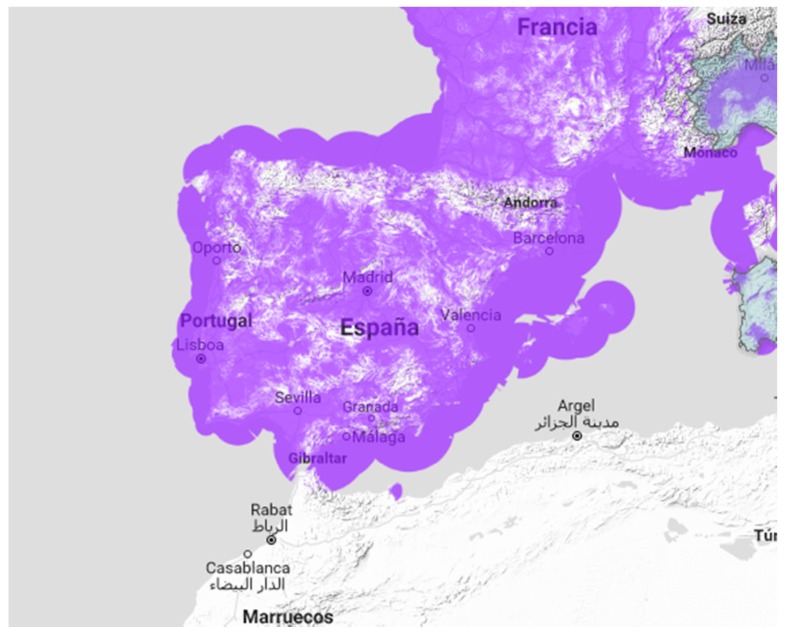
Coverage of the SIGFOX network in Spain [8].

The WSN can be classified depending on their communication link distances. On the one hand, there are WSN focused on short range communication (i.e., about 100 m or less), integrated by networks based on body area and/or buildings area. In this context, many researchers have been paying attention to the integration of these WSN for healthcare applications to monitor physiological (i.e., pulse oximetry, respiration rate, temperature, etc.) and physical behavior (i.e., limb movements, posture, and muscular activity) [3]. Typically, these short range WSN are based on Bluetooth, IEEE 802.15.4 and/or ZigBee communication standards [4]. On the other hand, there are WSN that require long communication range, such as the urban networks needed for the deployment of Smart Cities [5]. Traditionally, one solution to fulfill these long distance radio links was to use the mobile network operators (MNO). However, with the MNO network, each sensor node must have a subscriber identity module (SIM) card. This SIM-card increases the sensor node unit price and, therefore, it makes difficult the massive deployment of long range WSN. To overcome this drawback, low-power wide-area networks (LPWAN) are been developed to connect devices that require low bandwidth, low cost and high power efficiency [6]. LPWAN constitute a very recent machine to machine (M2M) solution with great projection in urban scenarios. Most network operators deploy their own base station infrastructure devoted to sensor node connectivity. In these networks, underlay wireless networking infrastructure offers connectivity and data transport between sensor nodes and a base station. Also, these base stations are connected by means of the operator core network to an operator gateway, which finally forwards sensor data to cloud servers on the Internet. With these networks, it is possible to cover a medium size city with a few number of base stations. Two alternatives stand out over the others; LoRa and SIGFOX [7]. Both solutions operate at sub-GHz ISM band. However, whereas LoRa is based on a spread spectrum technology, SIGFOX consists of a narrowband technology. The business model of both technologies is also different. LoRa specifications are open and the LoRa network must be developed by oneself. On the contrary, SIGFOX network is owned by SIGFOX Company, which is in charge of the deployment of their network. In this case, each sensor node must be subscribed. Spain is one of the first countries where the SIGFOX network has been deployed and it is currently implemented across most of the country, as shown in Figure 1.

The GPS trackers are not new devices. Nowadays several commercial alternatives to GPS tracking can be found in the market. On the one hand, a variety of applications for this particular purpose (APPs) can be downloaded and installed to a smartphone, such as *Life360* [9] and *Kid-Control* [10], among others. On the other hand, specific GPS tracking devices are available in the market to locate our beloved ones, pets or vehicles, such as *pocketfinder* [11], *tractive GPS* [12], etc. In all this cases, two drawbacks arise. Firstly, the use of the mobile network, with the resulting subscription cost that may be non-cumulative in some applications. Secondly, the lack of wearability and comfort due to the non-integration of the consumer electronics with an outfit. In fact, bespoke or mass produced body-worn solutions are key to increase wearability and comfort, an essential feature in applications such as the tracking of children or people with mental illness.

To the best knowledge of the authors, this is the first time a fully integrated wearable GPS tracking system based on SIGFOX protocol is presented. In this paper, the overall system is designed, programmed, and integrated with textile jeans substrate and tested in a real scenario (an adult wearing jean trousers including the on-body wireless system). Besides the GPS tracking smart textile functionality, it is mandatory to evaluate the amount of electromagnetic radiation absorbed by the body tissues for health safety reasons. This issue has been addressed through the specific absorption rate (SAR) assessment of the radiation system elements (i.e., wearable antenna), according to the corresponding international standards.

The remainder of the paper is organized as follows. In Section 2, the system architecture and wireless wearable sensor node are described. Specifically, the proposed smart textile tracking system is explained in terms of the GPS receiver, the microcontroller, the SIGFOX transmitter and the wearable antenna. In addition, the proposed implemented remote server and the visualization tools are presented. In Section 3, the overall system field test results are shown. Finally, in Section 4, the main results of the paper are discussed and concluded.

## 2. Materials and Methods

### 2.1. System Architecture

**Figure 2:**
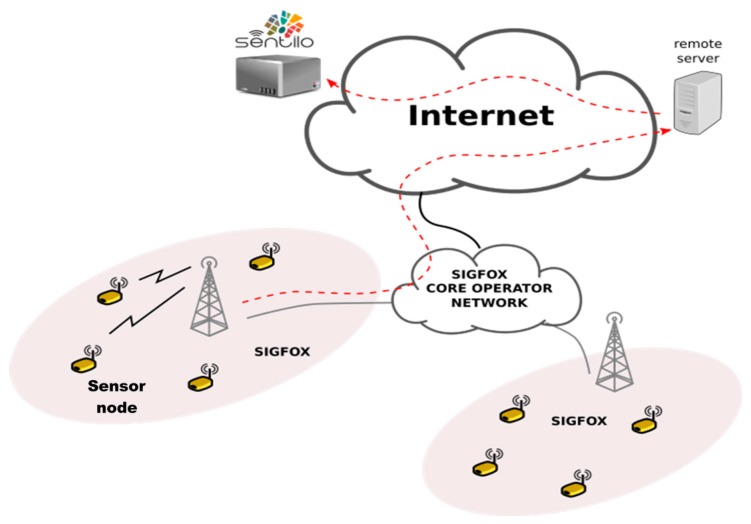
System Architecture.

As it is shown in Figure 2, the system architecture interconnection uses an LPWAN to transport data from sensor nodes to the final communication end-point hosted on the Internet. Specifically, in this system, the SIGFOX Network Operator has been used. SIGFOX is a leading operator of LPWAN with proprietary technology that offers an end-to-end connectivity solution, and has an extensive deployment in the Spanish territory. This operator deploys the proprietary base stations and connects them to the backend servers using an IP-based network. The end devices connect to these base stations using ultra narrow band (UNB) modulation technology. With this modulation technique, SIGFOX employs the bandwidth efficiently and experiences very low noise levels, resulting in a high receiver sensitivity, ultra-low power consumption, and inexpensive antenna design. All these benefits come at an expense of a maximum throughput of only 100 bps. The number and size of messages over the uplink are limited to 140 messages of 12-byte per day to conform to the regional regulations on use of license-free spectrum. Radio access link is asymmetric, allowing transmission of a maximum of only 4–8 bytes per day over the downlink from the base stations to the end devices.

The SIGFOX network provides a backend service where all network devices’ messages are received and the client/customer can retrieve data from their own devices through using a REST API. Operation through the REST API means that the client needs to poll the backend server for new messages, which is not very efficient. SIGFOX also provides an alternative mechanism to retrieve sensor data from backend servers. This mechanism is a callback function to be invoked for every message received on the backend. The callback can be done through email, where the data is sent by email to the destination, or through an HTTP call to a client/customer-owned server.

In this work, we use the HTTP callback mechanisms at the SIGFOX backend server to publish sensor data to our server. When sensor data is received at the backend server, it is encapsulated, with no further processing, over an HTTP message. Also, some metadata collected by the backend server and associated to this data can be sent in this HTTP message: sensor identification (id), reception time, signal-to-noise ratio (SNR), received signal strength indicator (RSSI) and a sequence number managed by the backend server.

The ultimate goal is to publish sensor data to the SENTILO platform. SENTILO is an open source sensor management platform developed to collect, exploit and disseminate information generated by sensors deployed in a city. The main goal of the SENTILO platform is to make it easy for cities to integrate data from different sensors and facilitate Smart City deployments. SENTILO platform provides a REST API that allows customers to use publish/subscription mechanisms. Specifically, it provides operations to publish, retrieve and delete data. The information transmitted to the SENTILO platform uses the JSON format.

In order to exploit efficiently the low bandwidth provided by the SIGFOX network, data generated by sensors have to be appropriately coded, and JSON format is not suitable to achieve this level of efficiency. The solution we propose is an intermediate server located on the Internet, namely a remote server. It receives raw sensor data sent by the SIGFOX backend using the HTTP callback mechanism. Sensor data is encoded using standard algorithms, such as IEEE 754 for floating point representation of numerical float values, or appropriate encoding algorithms for GPS coordinates. The sensor data is received, processed and properly formatted using the JSON standard, and a new HTTP message is generated to publish processed sensor data on the SENTILO platform, using the SENTILO REST API.

### 2.2. Wireless Wearable Sensor Node

**Figure 3:**
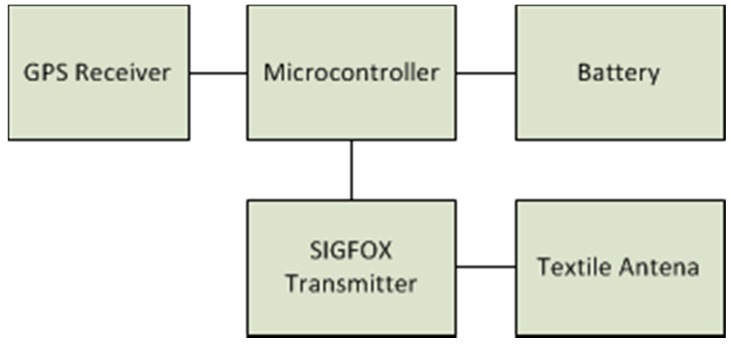
Block diagram of the proposed wireless wearable sensor node.

Figure 3 illustrates the block diagram of the proposed wireless wearable sensor node (WWSN). The main part (core) of the WWSN is the microcontroller. This block receives the location data from the GPS receiver by means of the NMEA0183 frame. This information is codified into the microcontroller and transmitted to the cloud by using the SIGFOX transmitter, which is connected to a specific textile antenna to efficiently radiate the information.

#### 2.2.1. GPS Receiver

**Figure 4:**
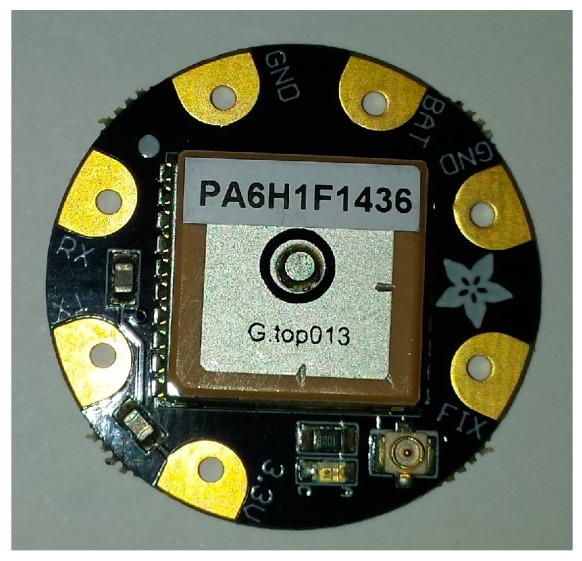
Flora GPS Module.

Figure 4 shows the Flora GPS Module, a wearable printed circuit board (PCB) based on a MTK3339 chipset. This chipset is able to track up to 22 satellites on 66 channels with a receiver acquisition and tracking sensitivity range of −145 dBm and −165 dBm, with a power supply between 3.0 V and 4.3 V, respectively. A GPS antenna is also included in this chipset and it will be the GPS antenna used in this work. The power consumption of the module is lower than 25 mA during navigation with an updated maximum frequency of 10 Hz. Besides the MTK330 chipset, the Flora GPS module also includes a ferrite, a decoupling capacitor for the supply (V_CC_) line, a U.FL connector for external antenna and an LED indicator, which blinks once per second during the searching phase and once every fifteen seconds during the tracking phase. The communication between the GPS module and the microcontroller is done by the UART (RX and TX pins). The GPS location is sent to the microcontroller using the NMEA 0183 protocol. This module has been selected mainly because its PCB is already prepared to be stitched on to any textile substrate.

**Figure 5:**
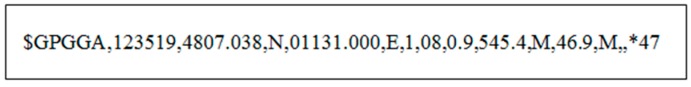
Example of NMEA 0183 GPS frame.

Figure 5 shows an example of the NMEA 0183 frame sent from the GPS module to the microcontroller. It starts with the $ character, then the GP characters are used to indicate that the device is a GPS module. After that, GGA denotes that the information corresponds to a GPS fix data time and position. The rest of characters are devoted to the send the information (time, latitude, longitude, number of satellite). Further information about the NMEA 0183 protocol can be found in [13].

#### 2.2.2. Microcontroller

**Figure 6:**
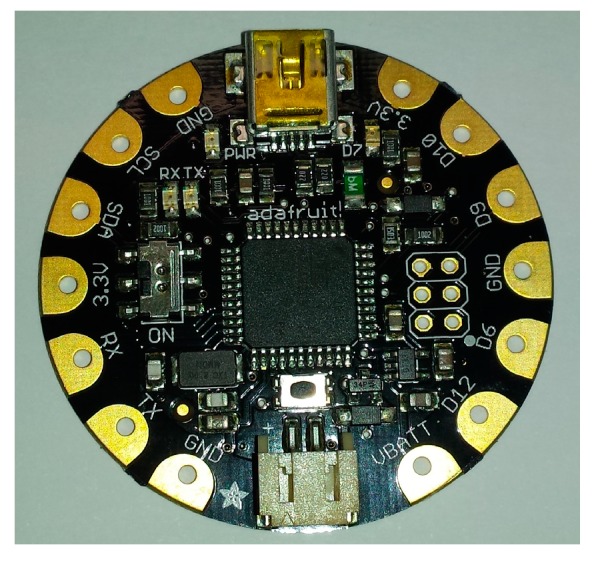
Flora Microcontroller.

The main part of the WWSN is the Adafruit Flora microcontroller V 1.0a, shown in Figure 6. The device is based on a RISC ATMEL MEGA 32U4 microcontroller which controls the sensor node, receives the NMEA 0183 location from the GPS module, codifies the GPS location and supplies this information to the SIGFOX module to be transmitted.

The module consists of a round printed circuit board of 45 mm diameter and it contains the ATMEL MEGA 32U4 chip with a 3.3 V regular voltage, a reset button and some LEDs to indicate if the module is supplied, receiving or transmitting data. The module can be supplied by means of the onboard polarized 2 JST battery connector or by the onboard mini B USB connector and it is programmed by means of a modified version of Arduino IDE. Like in the GPS module, this device has been selected because it is already prepared to be stitched on to the textile, increasing the wearability of the overall system.

The microcontroller is connected to the GPS module using the UART port that uses the D0 and D1 terminals and to the SIGFOX transmitter by means of an additional UART which requires the D10, D9 terminals. A 3.7 V battery is connected to the JST connector in order to supply all the system.

**Figure 7:**
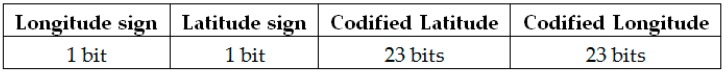
GPS Information to be transmitted by SIGFOX.

The developed firmware reads the NMEA 0183 information from the GPS module and sends this information to the SIGFOX network. However, in the SIGFOX protocol the maximum message payload data corresponds to 12 bytes and, therefore, the information to be transmitted should be compacted as much as possible. In order to do that, a codification algorithm has been implemented in the microcontroller [14]. With this algorithm, only 6 bytes are required to transmit the GPS location with an accuracy of within 2 m. Specifically, the two more significant bits are used to send the latitude and longitude signs. For each codified latitude and longitude value, 23 bits are required, as shown in Figure 7.

**Figure 8:**
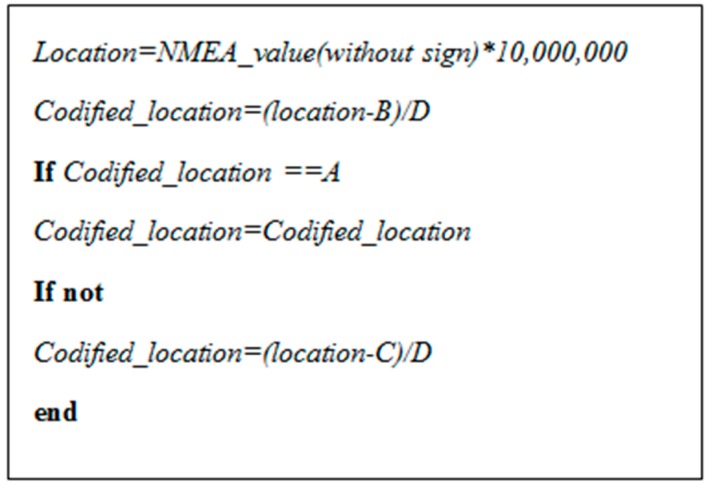
Pseudocode algorithm for latitude and longitude codification.

Figure 8 and Table 1 show the pseudocode and reference values used to obtain the codified latitude and longitude. The unsigned latitude/longitude value obtained from the NMEA 0183 GPS frame are multiplied by 10,000,000 in order to use an integer number. This value is codified by subtracting *B* and dividing by *D*. If this value corresponds to the reference value *A*, the codified algorithm ends. If not, the codified value is calculated by subtracting *C* and dividing by *D*. Once the codified latitude and codified longitude are obtained, the GPS information is ready to be transmitted according to Figure 7.

#### 2.2.3. SIGFOX Transmitter

**Figure 9:**
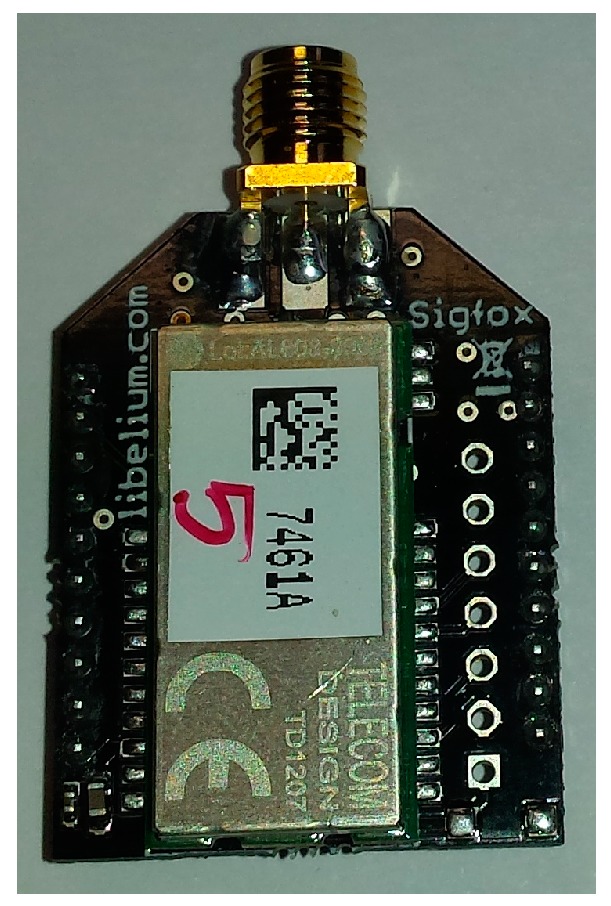
SIGFOX transmitter.

The SIGFOX gateway module is based on the high-performance, low-current SIGFOX TD1207. In a 25 pin land grid array package, the gateway includes a 32-bit ARM cortex-M3 baseband processor, an AES encryption engine, a DMA controller, several GPIOs, low energy UART and an RF front-end, as shown in Figure 9.

With a power supply of 3.3 V, the transceiver offers a sensitivity of −126 dBm and a maximum output power of +14 dBm, which allows for achieving long-distance communication at 868 MHz frequency. The current consumption of the module is lower than 16 mA in the reception mode and 37 mA with a power transmission of 10 dBm. The RF antenna output presents a single-ended 50 Ω impedance port which simplifies the antenna matching network. The SIGFOX transmitter is connected to the WWSN microcontroller by means of the low energy UART and the controller by using the HAYES commands [15]. The message sent is received by the SIGFOX backend and it is automatically forwarded to the remote server to decode the information received.

#### 2.2.4. Textile Antenna

**Figure 10:**
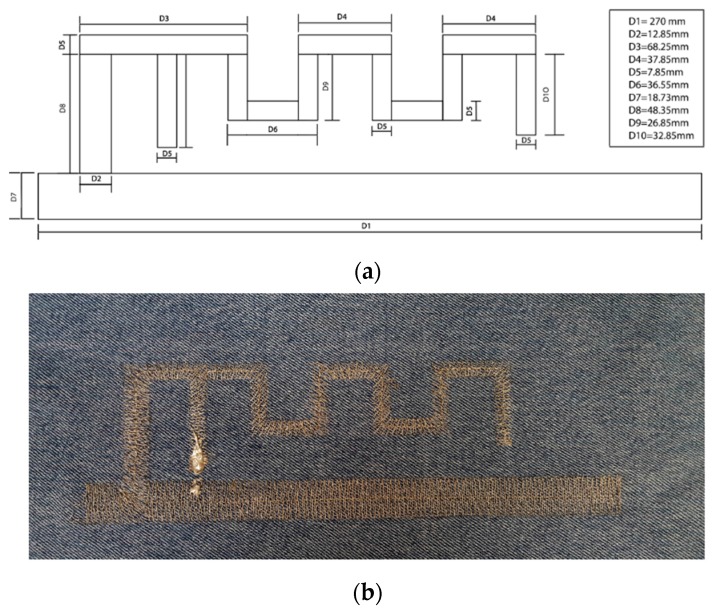
(**a**) Meandered inverted-F antenna MIFA prototype and dimensions; (**b**) Manufactured embroidered 868 MHz MIFA on a commercial jeans substrate.

**Figure 11:**
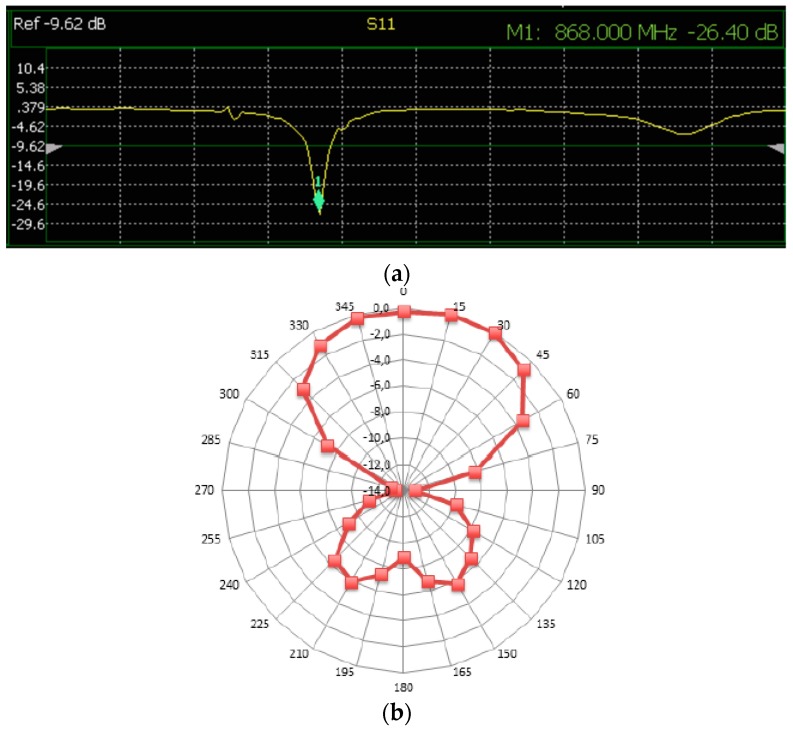
MIFA experimental results: (**a**) Return loss; (**b**) Realized gain diagram pattern at 868 MHz.

The proposed wearable SIGFOX antenna topology consists of a meandered inverted-F antenna (MIFA), as depicted in Figure 10. The main reason for selecting this topology is the size reduction in comparison with other antennas such as conventional monopoles or dipoles, by preserving a good antenna parameters performance. This issue is critical at the involved SIGFOX operation frequency (ISM 868 MHz) in order to locate a practical embroidered antenna on the outfit. The proposed MIFA has been designed and simulated by means of the commercial full 3D electromagnetic *CST Microwave Studio 2016* and tested to assess its performance in: return loss, radiation pattern, gain and efficiency. The antenna is embroidered on a standard jeans textile substrate with 1 mm thickness and the following electrical properties: dielectric constant εr = 1.7; loss tangent tan δ = 0.025, two key parameters to determine the efficiency and losses of the designed antenna. The conductor yarn corresponds to a commercial Shieldex 117/17 dtex 2-ply and it is composed of 99% pure silver plated nylon yarn 140/17 dtex with a linear resistance of <30 Ω/cm. Figure 10a defines the dimensions of the final prototype after a design tuning process for a 50 Ω input feeding. The overall size of the MIFA corresponds to 75 × 270 mm^2^. A Singer Futura XL-550 embroidery machine has been used to manufacture the wearable antenna, using a satin fill pattern to stitch the conductive area. The implemented jeans antenna is shown in Figure 10b. The antenna performance has been measured in an RF diagnostic chamber R&S DST200 (anechoic chamber) by means of a N9916A FieldFox microwave analyzer operating as vector network analyzer. Figure 11a depicts the measured S11 parameter (return losses). An excellent behaviour at the antenna operational frequency is observed (S11 = −26.4 dB @ 868 MHz), since the standard accepted value is S11 < −10 dB. In addition, a manual 3D positioner has been used to measure the radiation pattern gain by considering an angle step of 15°. Figure 11b illustrates the normalized radiation pattern (XZ plane) of the MIFA. As observed, a reasonable directive pattern is achieved. The antenna directivity and radiation efficiency correspond to D = 1.93 dBi and η = 62.6%, respectively.

**Figure 12:**
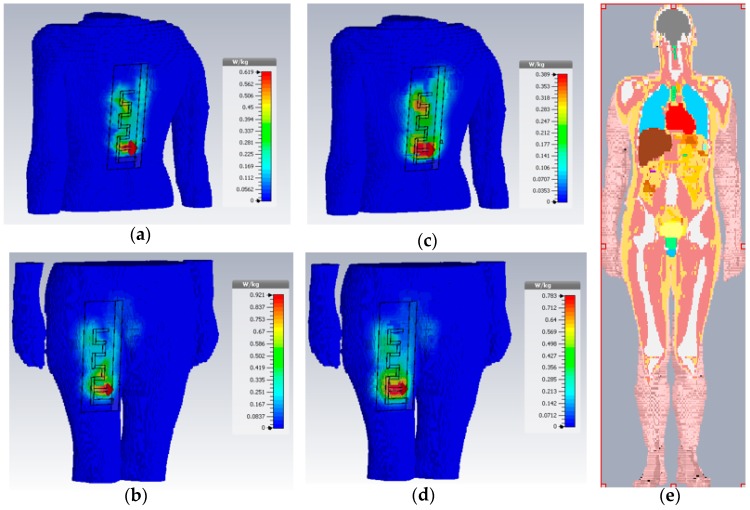
On-body jeans MIFA antenna 3D specific absorption rate (SAR) distribution at 868 MHz averaged over 1 g (**a**,**b**) and 10 g (**c**,**d**) tissue. The antenna has been located in the back and leg of a 38 year male voxel model called Gustav (**e**).

Besides MIFA electronic performance, body-worn antennas must satisfy the electromagnetic fields exposure limits according to the current legal regulations [16]. This issue is addressed by determining the specific absorption rate (SAR), defined as the electromagnetic energy absorbed by human biological tissue when exposed to the antenna radiation [17]. The allowed general public exposure limits of SAR are: 1.6 W/Kg and 2 W/Kg averaged over 1 g and 10 g tissue, respectively. Several standard SAR simulations have been carried out by considering realistic human voxel models according to the regulators (IEEE/IEC 62704-1). Those models are based on more than 50 human tissues, including their realistic non-homogeneous electric properties [18]. As an example, Figure 12 shows the on-body jeans MIFA antenna 3D SAR distribution at 868 MHz averaged over 1 g and 10 g tissue for the Gustav male model (38 years old, 176 cm size, 69 Kg mass) in different body locations. Moreover, an additional voxel including the Donna female model (40 years old, 176 cm size, 79 Kg mass) has been tested. In both cases the MIFA has been located in the leg of the models (trousers). Table 2 summarizes all the maximum SAR values obtained in the two cases under study. It is fundamental to remark that in all of the analysed cases, the peak SAR values are below the maximum allowed limits. Moreover, in the worst case (woman) the regulation compliance is satisfied with a safe margin of more than 50% over the 1 g tissue threshold and 75.7% over the 10 g tissue, respectively.

### 2.3. Remote Server

The proposed remote server is used in order to receive the raw sensor data sent by the SIGFOX network. After decoding the GPS latitude and longitude, it generates a new HTTP message with the JSON standard and, finally, it sends the information to the data viewer (SENTILO). This server is implemented using Linux OS and the reception of the SIGFOX HTPP message is achieved via inetd daemon which calls a set of python scripts to process raw sensor data, to binary encode sensor data appropriately and, finally, to generate a new HTTP message to the SENTILO platform with JSON representation by means of SENTILO REST API.

### 2.4. SENTILO Open Source Data Viewer

In order to represent the position of the sensor node, the open source data viewer SENTILO has been used. SENTILO is an open source platform developed by the Barcelona City council to fit the requirements of a Smart City and share information between various systems. With this system, the reliance on specific technologies is reduced. The platform is a Java system based on Redis and includes a REST API, web application management, storing data in a relational database and agent for notifying alerts.

**Figure 13:**
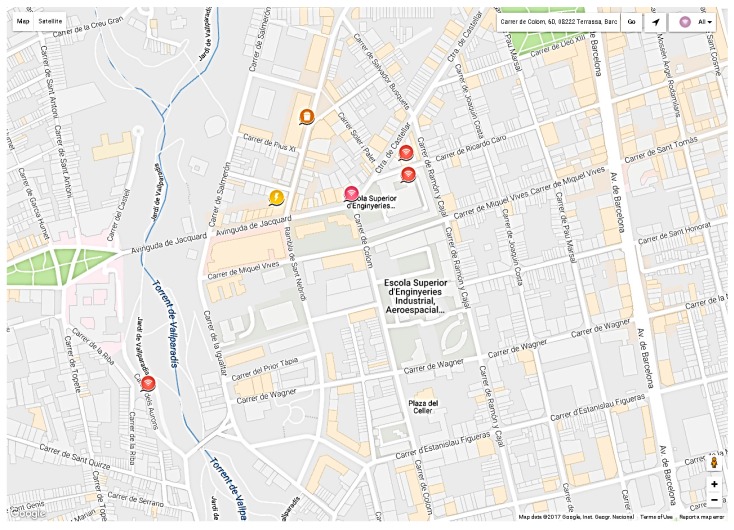
Universal viewer of the SENTILO platform.

Two different viewer modes are present in SENTILO: the universal viewer, for static sensors, and the route viewer for motion sensors. Figure 13 shows the universal viewer of the SENTILO platform, based on Google Maps. In this map the sensors are located and the measurements values of each sensor are shown. Since we are developing a tracking system, the route viewer mode is used in this application. It should be remarked that in the route viewer only the last 20 points are displayed for each sensor, which is enough for this application.

In order to send the information to the SENTILO platform, the API defines the HTTP protocol method. In particular, the location information of each sensor is sent to SENTILO by means of HTTP POST with a JSON format.

## 3. Results

**Figure 14:**
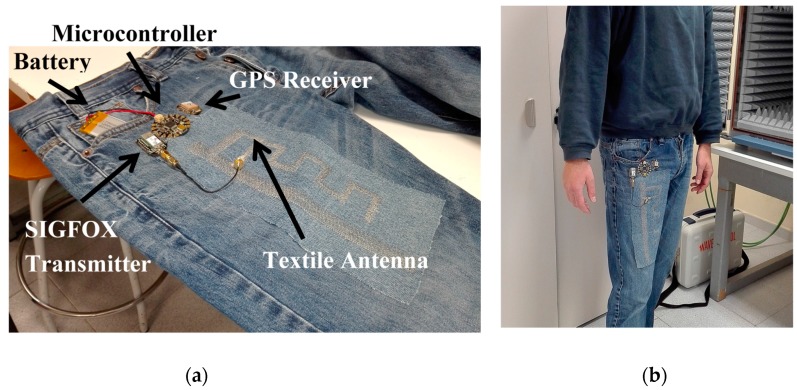
(**a**) Details of the proposed wireless wearable sensor node; (**b**) On-body wearable system.

Figure 14 shows a photograph of the proposed wireless wearable sensor node. As can be observed, the prototype has been implemented on the right leg of usual jean trousers. The electrical connections between the different modules (GPS receiver, microcontroller and SIGFOX transmitter) have been implemented by stitching conductor yarn on the jeans.

**Figure 15:**
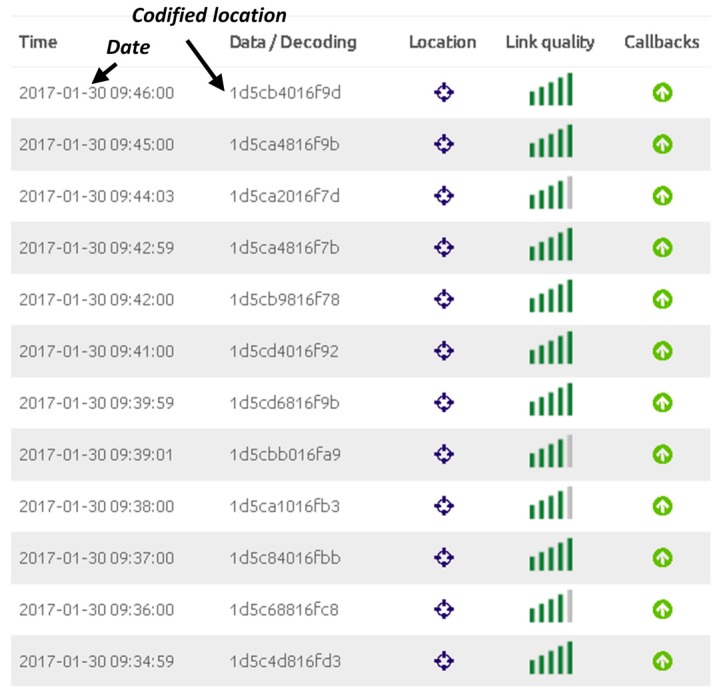
Screenshot of SIGFOX Backend.

**Figure 16:**
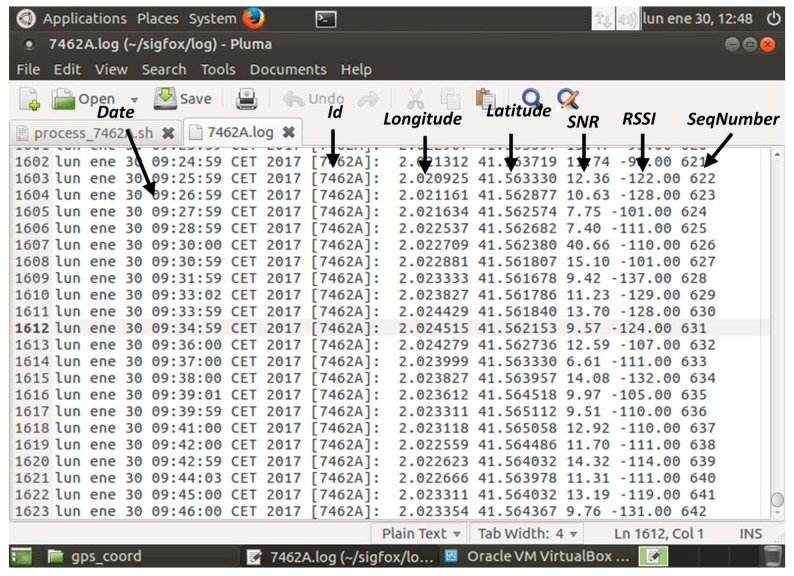
Details of the information send from the remote server to SENTILO.

The proposed wearable tracking system has been tested in Terrassa, province of Barcelona. Terrassa (with a population of 215,000 inhabitants) bets on its development as a Smart City. Indeed, the city has developed a platform of smart sensors based on SENTILO. The system was evaluated by one person walking around the Campus of the Universitat Politècnica de Catalunya in Terrassa without any incident. The wireless wearable sensor node was configured to transmit the GPS coordinates every minute. Concerning the overall system consumption, it depends on the operation mode. The maximum current consumption is <100 mA and it is produced during the transmission mode. As a reference, for a battery with a nominal capacity of 1200 mAh and assuming the more stringent mode (continuous transmission), a battery lifetime of 12 h is expected. A trade-off between battery lifetime and tracking resolution appears. In practice, for mobility on foot tracking system a good compromise can be achieved by sending the location every few minutes. Figure 15 shows the SIGFOX backend for a one minute transmission time. As it is observed, each minute a message with the latitude codifications is sent by the wearable sensor node and received in the SIGFOX backend. The message is directly forwarded to the developed remote server to decode the latitude and longitude and additional information. Once the information is decoded, it is transmitted to SENTILO, as depicted in Figure 16.

**Figure 17:**
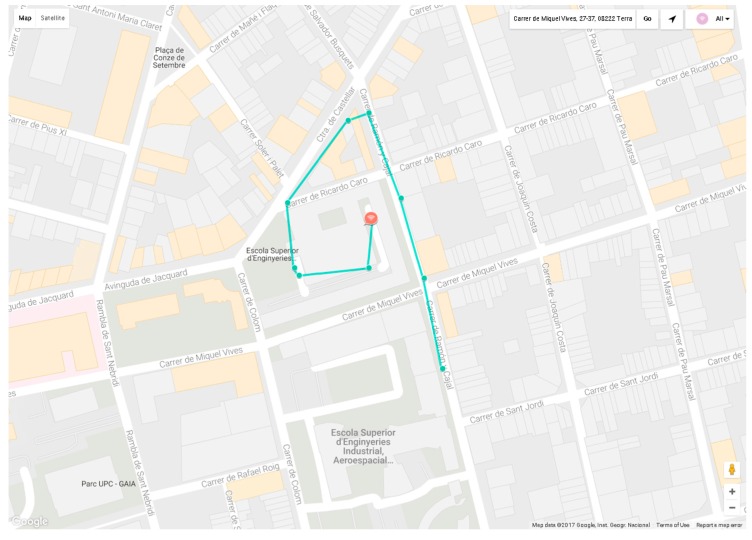
Screenshot of SENTILO route viewer.

Finally, the location of the wearable wireless sensor node is shown in SENTILO route viewer, as detailed in Figure 17. Note that SENTILO route viewer shows the last ten locations of the WWSN node and draws the path followed by the node. In this example, the test route was around the Terrassa School of Industrial, Aerospace and Audiovisual Engineering, located in the center of the town.

## 4. Discussion and Conclusions

In this work, an alternative wearable tracking system based on the low-power wide area network has been developed. The system consists of a GPS receiver and SIGFOX transmitter integrated with a textile substrate. In order to use the SIGFOX network to transmit the latitude and longitude coordinates, a specific codification algorithm has been used. In order to enhance the wearability of the system, a customized UHF antenna on jeans fabric has been designed and developed. The impact of the electromagnetic radiation absorbed by the human body has been evaluated and it is under the limits defined in the electromagnetic exposure international regulations, guaranteeing the health safety of the system. The coded coordinates have been decoded in a specific developed remote server and sent to the open source data viewer SENTILO. The functionality of the system has been experimentally evaluated by means of a field test in Terrassa.

The proposed system enhances the wearability and reduces the cost with regard to other alternatives. However, in order to increase the wearability of future nodes even more, work is in progress to integrate all the electronic components in a single wearable PCB and develop an additional GPS textile antenna. Moreover, other aspects such as the washing process and wear-out impact should be addressed.

The results pointed out that a low-cost wearable tracking system based on LPWAN can be integrated into textile with a successful result. This alternative could be an interesting method for applications such as tracking children or people with mental illness.

## Figures and Tables

**Table 1:** Reference Values used to obtain the codified latitude and longitude.

Parameter	Latitude	Longitude
***A***	8.333.333	8.372.093
***B***	18	2
***C***	53	107
***D***	108	215

**Table 2:** Computed SAR values for the jeans MIFA implemented in trousers under test @ 868 MHz.

Voxel Model	Peak SAR 1 g Tissue (W/Kg)	Peak SAR 10 g Tissue (W/Kg)
38 year male	0.619	0.389
40 year female	0.998	0.389

## References

[1] Chen S., Zhao C., Wu M., Sun Z., Zhang H., Leung V.C.M. (2016). Compressive network coding for wireless sensor networks: Spatio-temporal coding and optimization design. Comput. Netw..

[2] Piñuela M., Mitcheson P.D., Lucyszyn S. (2013). Ambient RF energy harvesting in urban and semi-urban environments. IEEE Trans. Microw. Theory Tech..

[3] Ko J., Lu C., Srivastava M.B., Stankovic J.A., Terzis A., Welsh M. (2010). Wireless Sensor Networks for Healthcare. Proc. IEEE.

[4] Baronti P., Pillai P., Chook V.W.C., Chessa S., Gotta A., Hu Y.F. (2007). Wireless sensor networks: A survey on the state of the art and the 802.15.4 and ZigBee standards. Comput. Commun..

[5] Gomez C., Paradells J. (2015). Urban Automation Networks: Current and Emerging Solutions for Sensed Data Collection and Actuation in Smart Cities. Sensors.

[6] Raza U., Kulkarni P., Sooriyabandara M. (2017). Low Power Wide Area Networks: An Overview. IEEE Commun. Surv. Tutor..

[7] Nolan K.E., Guibene W., Kelly M.Y. An evaluation of low power wide area network technologies for the Internet of Things. Proceedings of the IEEE 2016 International Wireless Communications and Mobile Computing Conference (IWCMC).

[8] SIGFOX Company. https://www.sigfox.com.

[9] Life360 App. https://www.life360.com.

[10] Kid-Control App. http://kid-control.com.

[11] PocketFinder GPS Trackers. http://pocketfinder.com.

[12] Tractive GPS. https://tractive.com/en.

[13] National Marine Electronics Association (2008). NMEA 0183 V4.0 Stand.

[14] Aguirre X., Fernandez-Garcia R. (2016). Proyecto de desarrollo de un localizador GPS.

[15] TDNext (2016). RF Modules AT Reference Manual.

[16] Madjar H.M. Human radio frequency exposure limits: An update of reference levels in Europe, USA, Canada, China, Japan and Korea. Proceedings of the IEEE 2016 International Symposium on Electromagnetic Compatibility, EMC EUROPE.

[17] IEEE Standards Coordinating Committee 28 on N.-I.R.H., Institute of Electrical and Electronics Engineers, IEEE-SA Standards Board (2003). IEEE Recommended Practice for Measurements and Computations of Radio Frequency Electromagnetic Fields with Respect to Human Exposure to Such Fields, 100 kHz–300 GHz.

[18] CST Company. www.cst.com.

